# Role of Retinoids and Their Analogs in the Treatment of Cutaneous T-cell Lymphoma: A Systematic Review

**DOI:** 10.7759/cureus.69318

**Published:** 2024-09-13

**Authors:** Shriya Ayuthu, Yashkumar D Chauhan, Amna A Mirza, Moyal Z Saad, Parikshit Bittla, Sai Pavitra Paidimarri, Tuheen Sankar Nath

**Affiliations:** 1 Internal Medicine, California Institute of Behavioral Neurosciences & Psychology, Fairfield, USA; 2 Medicine, California Institute of Behavioral Neurosciences & Psychology, Fairfield, USA; 3 Surgical Oncology, California Institute of Behavioral Neurosciences & Psychology, Fairfield, USA

**Keywords:** bexarotene, cutaneous t cell lymphoma, mycosis fungiodes, oral retinoid, sezary syndrome, topical retinoids

## Abstract

Biologically active derivatives of vitamin A, known as retinoids, can be used to treat cutaneous T-cell lymphomas. Retinoids and their analogs can modulate cell proliferation, differentiation, and apoptosis, and alter the immune response. This study systematically evaluated the safety, efficacy, and tolerability of retinoids for the treatment of cutaneous T-cell lymphomas and considered its limitations, dosing-side effects, and technique with the intent to provide valuable insights for clinicians and patients regarding the treatment of cutaneous T-cell lymphomas with the retinoids. The literature search is conducted using Preferred Reporting Items for Systematic Review and Meta-analyses (PRISMA) criteria yielding 16 relevant articles. This study explored the different facets of the role of retinoids in the treatment of cutaneous T-cell lymphomas. In conclusion, the available studies and research have shown that retinoids play an important role in the mild and early stages of cutaneous T-cell lymphoma. However, further investigation is required to explain the mechanism of action of retinoids and the impact of their side effects in patients with cutaneous T-cell lymphoma.

## Introduction and background

Cutaneous lymphomas are heterogeneous groups of extranodal non-Hodgkin lymphomas limited to the skin at diagnosis [1]. The primary cutaneous lymphomas are first classified by the European Organization for Research and Treatment of Cancer (EORTC) based on their indolent, intermediate, and aggressive clinical behavior, providing the clinician comprehensive information on staging, the preferred mode of treatment, clinical behavior, and prognosis. However, it included very few types of cutaneous lymphomas. In 2005, the European Organization for Research and Treatment of Cancer (EORTC), in collaboration with the World Health Organization (WHO), provided a consensus classification for cutaneous lymphomas [2], which was revised in 2016 after incorporating different cutaneous B-cell lymphomas and cutaneous T-cell lymphomas. In 2018, the updated version of the WHO-EORTC classification of cutaneous lymphomas was published. Cutaneous T-cell lymphoma is commonly observed compared to cutaneous lymphomas. The main variants of cutaneous T-cell lymphoma are mycosis fungoides (MF) and Sezary syndrome (SS). The incidence of primary cutaneous T-cell lymphomas is currently 8.55 per million based on Surveillance, Epidemiology, and End Results registry data (SEER), with mycosis fungoides (MF) having the highest incidence of 5.42 and Sezary syndrome (SS) with an increased number of cases [3]. 

The pathophysiology of cutaneous T-cell lymphoma is poorly understood. The potential agents include infectious agents, ultraviolet (UV) light, and occupational exposures. Through large-scale mutational genomic profiling-potential oncogenes and tumor suppressor genes like CARD11, CCR4, TP53, NF-kB, and genes related to the JAK signaling pathway, epigenetic modifications, and histone acetylation were identified. In mycosis fungoides, the malignant cells are clonal effector memory T-cells, which are mainly present in the skin, explaining the skin-bound nature of the lesions. In contrast, Sezary syndrome consists of central memory T-cells with lymph node-homing molecules like CCR7 and L-selectin, resulting in an erythrodermic phenotype peripherally, with blood and lymph node involvement centrally [4,5].

The management of MF/SS depends on the stage of the disease and systemic involvement. In the patients of MF/SS, the therapy is mainly palliative, giving priority to the quality of life. However, the principle behind allogeneic stem cell transplantation in advanced disease and long-term remission after skin-directed therapy is curative [6,7]. The management options and treatment modalities included are described in Table 1.

**Table 1:** The treatment options available for cutaneous T-cell lymphoma. CHOP regimen: cyclophosphamide, hydroxy doxorubicin, oncovin (vincristine) [7].

Skin directed therapies	Systemic therapies
Topical corticosteroids	Retinoids (including bexarotene)
Topical options without recommendations- retinoids, calcineurin inhibitors	Interferon-γ
Ultraviolet phototherapy	Combinations of skin-directed therapies
Photodynamic therapy	Chemotherapy-mono chemotherapy (gemcitabine, pegylated liposomal doxorubicin), CHOP regimen (cyclophosphamide, hydroxy doxorubicin, oncovin (vincristine), prednisone), others-chlorambucil and methotrexate
Total skin electron beam therapy	Targeted immunotherapy-alemtuzumab, mogamulizumab, brentuximab vedotin
Localised radiotherapy	Extracorporeal photochemotherapy
Others-imiquimod, methotrexate, 5-flourouracil	Hematopoietic stem cell transplantation

In this article, we are undertaking a systematic review focusing on the role of retinoids and their analogs in treating cutaneous T-cell lymphomas. Our analysis compares the safety and efficacy of retinoids and their analogs in the treatment at different stages of cutaneous T-cell lymphomas. The review intends to provide valuable insights into clinical outcomes achieved with retinoids alone or combined with other drugs, gathering valuable evidence from relevant studies. Ultimately, this systematic review aims to advance the understanding of the treatment of cutaneous T-cell lymphomas, providing valuable guidance to healthcare providers for informed treatment decisions.

## Review

We used the Preferred Reporting Items for Systematic Reviews and Meta-Analyses (PRISMA) 2020 guidelines and principles to design this systematic review and share the outcomes.

Methods

Search-Strategy

We utilized research databases and search engines such as PubMed, Google Scholar, and Science Direct with appropriate keywords and Medical Subject Headings (Mesh) terms to find significant articles. We combined the main keywords using Boolean “AND” and “OR”. The number of articles identified in different databases is listed below in Table 2. The summary of the PubMed search strategy is mentioned in Table 3.

**Table 2:** Number of articles in various databases.

Databases	Articles
PubMed Mesh	75
Google Scholar	3970
ScienceDirect	3127

**Table 3:** Search strategy for PubMed. The articles included are from PubMed only.

PubMed search	Major mesh terms	Results
Concept 1	(("Lymphoma, T-Cell, Cutaneous/classification"(Majr) OR "Lymphoma, T-Cell, Cutaneous/complications"(Majr) OR "Lymphoma, T-Cell, Cutaneous/drug therapy"(Majr) OR "Lymphoma, T-Cell, Cutaneous/epidemiology"(Majr) OR "Lymphoma, T-Cell, Cutaneous/metabolism"(Majr) OR "Lymphoma, T-Cell, Cutaneous/mortality"(Majr) OR "Lymphoma, T-Cell, Cutaneous/prevention and control"(Majr) OR "Lymphoma, T-Cell, Cutaneous/therapy"(Majr))	4401
Concept 2	("Retinoids/administration and dosage"(Majr) OR "Retinoids/adverse effects"(Majr) OR "Retinoids/agonists"(Majr) OR "Retinoids/classification"(Majr) OR "Retinoids/metabolism"(Majr) OR "Retinoids/pharmacokinetics"(Majr) OR "Retinoids/pharmacology"(Majr) OR "Retinoids/standards"(Majr) OR "Retinoids/therapeutic use"(Majr) OR "Retinoids/toxicity"(Majr))	30186
Combined	The combined search strategy of keywords and mesh Keywords	75

Inclusion and Exclusion Criteria

We incorporated randomized controlled trials (RCTs), clinical trials, observational studies, and review articles published in English. Our review includes human studies focusing primarily on patients older than 45, which is relevant to our research question to understand recent advancements in this area comprehensively. We excluded animal studies, case reports, unpublished literature, and books that do not have open access. The inclusion and exclusion criteria are illustrated in Table 4.

**Table 4:** Inclusion and exclusion criteria. Inclusion and exclusion criteria for all the articles included in the study.

Inclusion criteria	Exclusion criteria
Papers published in English language only	Papers not published in the English language
Randomized clinical trials, meta-analysis, clinical trials, multicenter studies, observational studies, comparative studies, systematic reviews	Letters, animal studies, case reports, unpublished literature, books
Publications related to the query	Publications unrelated to the query
Studies that included humans	Studies that included animals
Studies that have open access	Studies that need to be purchased
Age included above 45	Excluded below 45

Results

**Figure 1:**
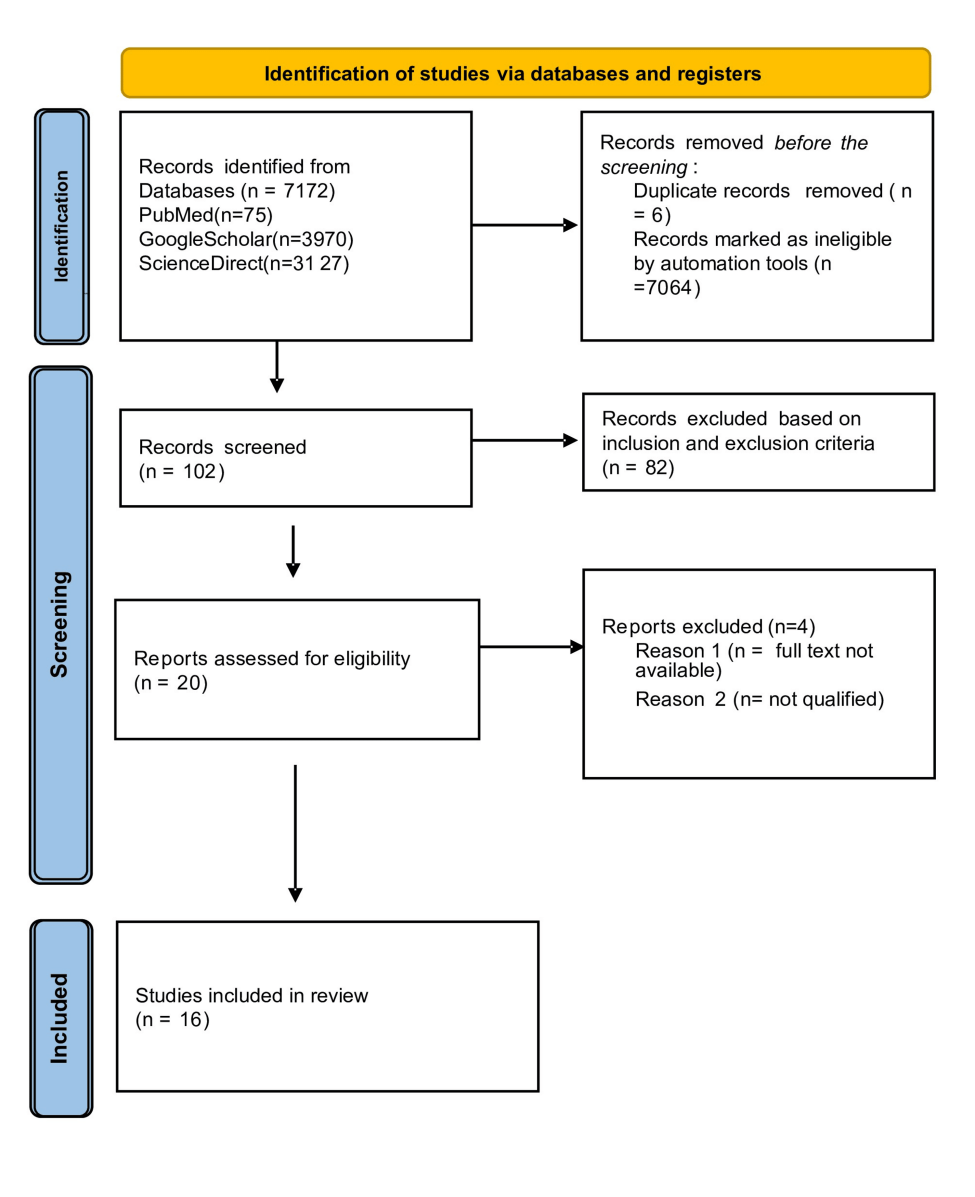
PRISMA flow chart. PRISMA: Preferred Reporting Items for Systematic Reviews and Meta-Analyses.

Two writers independently searched the databases for articles that met the inclusion and exclusion criteria. In cases of disagreement, a third author was included to address them. We reviewed the titles and abstracts to select the articles that would meet the goals of this study. We identified 7172 articles from all the databases, including PubMed (n=75), Google Scholar (3970), and ScienceDirect (n=3127). After removing duplicates and ineligible articles using automated tools (free full text, humans, PubMed articles from 1985, past 10 years in Google Scholar, and 15 years in ScienceDirect) and applying inclusion and exclusion criteria, 7156 articles were rejected. Twenty articles were assessed for eligibility and relevance to the review with full-text availability. Sixteen articles were included in the review. The Preferred Reporting Items for Systematic Review and Meta-Analysis (PRISMA) flowchart is demonstrated in Figure 1.

Characteristics of Study

We have reviewed 16 articles, including eight retrospective studies, two cohort studies, one retrospective cohort study, one randomized clinical trial, two narrative reviews, one systematic review, and one open-label pilot study. In all these studies, different types of retinoids and combinations are studied for the treatment of cutaneous T-cell lymphoma. Table 5 displays the characteristics of all the articles included in our systematic review.

**Table 5:** Characteristics of different articles included in the study.

S. No.	Author name and year of publication	Title	Study name	Years	ORR	Sample size	Study duration	Results	Ref
1.	Duvic et al.	Phase 2 and 3 clinical trials of oral bexarotene (Targretin capsules) for the treatment of refractory or persistent early-stage cutaneous T-cell lymphoma	Randomized clinical trial	2001	54%	58	22 months	Phase 2 and 3 clinical trials of bexarotene were conducted to determine the efficacy of bexarotene in treating cutaneous T-cell lymphoma. 54% of patients at 300 mg/m^2^ daily doses exhibited good tolerance and efficacy. Side effects that are reversible and treatable are seen.	[8]
2.	Alhusayen et al.	Evaluation of alitretinoin for the treatment of mycosis fungoides and Sezary syndrome	Retrospective study	2021	40%	48	Five years	Early and late stages of a cutaneous T-cell lymphoma responded to alitretinoin as a primary and combined therapy effectively.	[9]
3.	Kaemmerer et al.	Alitretinoin in the treatment of cutaneous T-cell lymphoma	Retrospective study	2021	37.2%	35	Five years	Alitretinoin can be considered an alternative to Bexarotene, due to its favorable response, fewer side effects, and reduced cost.	[10]
4.	Amity-Laish et al.	Retinoic acid receptor agonist as monotherapy for early-stage mycosis fungoides: does it work?	Retrospective study	2019	-	35	Seven years	The effect of Retinoic acid receptor agents (Isotretinoin and Acitretin) was studied in early-stage cutaneous T-cell lymphoma with an overall good response rate.	[11]
5.	Cheeley et al.	Acitretin for the treatment of cutaneous T-cell lymphoma	Retrospective study	2013	59%	32	28 months	For early-stage cutaneous T-cell lymphoma, acitretin is well tolerated and effective either as monotherapy or a combination.	[12]
6.	Sokołowska-Wojdyło et al.	Polish lymphoma research group experience with bexarotene in the treatment of cutaneous T-cell lymphoma	Retrospective study	2016	81%	21	Five years	Bexarotene's overall response rate was 81.0%. The early-stage cutaneous T-cell lymphoma responded well compared to the late-stage. The significant side effects observed were hyperlipidemia, hypothyroidism, and a bleeding gastric ulcer.	[13]
7.	Rouanet et al.	Real-life use of bexarotene for T-cell cutaneous lymphoma management: efficacy and tolerance with low doses	Retrospective study	2022	71%	64	14 years	The best clinical response with Bexarotene was seen with a median dose of 300 mg/day.	[14]
8.	Apisarnthanarax et al.	Tazarotene 0.1% gel for refractory mycosis fungoides lesions: an open-label pilot study	Pilot label study	2004	58%	20	24 weeks	This study evaluated the efficacy and tolerability of topical tazarotene 0.1% gel as adjuvant therapy in refractory mycosis fungoides lesions.	[15]
9.	Beltran et al.	Low-dose bexarotene alone or in combination for the treatment of relapsed or refractory cutaneous T-cell lymphoma	Retrospective study	2009	69%	13	Six years	Bexarotene alone achieved a 66% ORR, Bexarotene/Interferon-γ with a 40% overall response rate, and Bexarotene/Phototherapy with a 100% overall response rate in patients with relapsed/refractory cutaneous T-cell lymphoma.	[16]
10.	Quereux et al.	Bexarotene in cutaneous T-cell lymphoma: a third retrospective study of long-term cohort and review of the literature	Retrospective study	2013	60%	32	10 years	With a long study duration, this study states that Bexarotene is well-tolerated with mild to moderate side effects.	[17]
11.	Weichenthal et al.	Response of rare variants of cutaneous T cell lymphoma (cutaneous T-cell lymphoma) To treatment with bexarotene. A prospective German DeCOG Trial	Cohort study	2013	37%	200	24 weeks	Bexarotene is a safe and effective treatment in non-classical mycosis fungoides and cutaneous T-cell lymphoma variants.	[18]
12.	Roccuzzo et al.	Time to next treatment and safety assessment in cutaneous‐T‐cell lymphomas: a retrospective analysis on patients treated with bexarotene and acitretin	Retrospective cohort study	2022	66-75%	82	Four years	This study confirms that Bexarotene and acitretin are effective agents against the early stages of Cutaneous T-cell lymphoma. Bexarotene has more side effects than acitretin but is used in more advanced diseases than acitretin, which is used in early disease.	[19]
13.	Hamada et al.	Safety and efficacy of bexarotene for Japanese patients with cutaneous T-cell lymphoma: real-world experience from post-marketing surveillance	Cohort study	2022	58%	267	Two years	Mycosis fungoides patients with the regular dose in combination with phototherapy show a favorable response to Bexarotene. There are decreased side effects in patients with a reduced dose cohort.	[20]
14.	Panchal et al.	The utility of bexarotene in mycosis fungoides and Sezary syndrome	Narrative review	2015	N/A	N/A	N/A	Bexarotene is safe and efficient for advanced refractory mycosis fungoides/Sezary syndrome with predictable dosage-dependent adverse effects. Bexarotene combined therapies are found to be effective.	[21]
15.	Huen et al.	The role of systemic retinoids in the treatment of cutaneous T-cell lymphoma	Narrative review	2015	N/A	N/A	N/A	Retinoids play an important role in treating all stages of Cutaneous T-cell lymphoma. Bexarotene is an Food and Drug Administration (FDA)-approved systemic drug therapy, especially for cutaneous T-cell lymphoma, but it requires extra medication to treat the associated side effects.	[22]
16.	Sokolowska-wojdylo et al.	Oral retinoids and rexinoids in cutaneous T-cell lymphoma	Narrative review	2013	N/A	N/A	N/A	This demonstrates that rexinoids are a reliable and effective treatment for cutaneous T-cell lymphoma, achieving positive responses in half of the patients with cutaneous T-cell lymphoma.	[23]

Quality Appraisal of Individual Studies

Two authors were involved in the quality appraisal of the selected articles using the following tools: (1) the Newcastle-Ottawa scale for observational studies, (2) the Cochrane risk of a bias assessment tool for RCTs, and (3) the scale for quality assessment of narrative reviews. They meticulously reviewed 21 articles for quality assessment. Sixteen were qualified as medium/high and included in the review. The RCT [8] was included after assessing for eligibility. Tables 6-9 details the chosen observational studies and narrative studies quality assessment.

**Table 6:** Cochrane risk of bias assessment for randomized clinical trial.

	Duvic et al. [8]
Adequately generated allocation sequence was ensured	+
Adequately concealed allocation sequence was ensured	+
Allocation was concealed adequately	+
Participants and staff were blinded to the intervention	+
Outcome evaluators are blinded to the intervention	+
Incomplete outcome data were adequately addressed	+
Reports of the study were free from the suggestion of selective outcome reporting	+
The study was free of other problems that could put it at risk of bias.	+

**Table 7:** Quality assessment for case-control studies. Newcastle-Ottawa scale is used for case-control studies.

Selection	Alhusayen et al. [9]	Kaemmerer et al. [10]	Laish et al. [11]	Cheeley et al. [12]	Sokołowska-Wojdyło et al. [13]	Rouanet et al. [14]	Apisarnthanarax et al. [15]	Beltran et al. [16]	Quereux et al. [17]
Case definition adequate	1	1	1	1	1	1	1	1	1
Representativeness of cases	1	1	1	1	1	1	1	1	1
Selection of controls	1	1	1	1	1	1	1	1	1
Definition of controls	1	1	1	1	1	1	1	1	1
Comparability									
Comparability of cases and control based on design or analysis	2	2	2	2	2	2	0	2	2
Exposure									
Ascertainment of exposure	2	2	1	2	1	1	2	1	1
The same method of ascertainment for cases and controls	1	1	1	1	1	1	1	1	1
Non-response rate	1	1	1	1	1	1	1	1	1
Score	10	10	10	10	10	10	8	10	10

**Table 8:** Quality assessment table for cohort studies. Newcastle-Ottawa scale is used for case-control studies.

	Weichenthal et al. [18]	Roccuzzo et al. [19]	Hamada et al. [20]
Selection			
Representativeness of the exposed cohort	1	1	1
Selection of the non-exposed cohort	1	1	1
Ascertainment of exposure	1	1	1
Demonstration that outcome of interest was not present at the start of the study	1	1	1
Comparability			
Comparability of cohorts based on the design or analysis	2	2	2
Outcome			
Assessment of outcome	1	1	1
Was follow-up long enough for outcomes to occur	1	1	1
Adequacy of follow-up of cohorts	1	1	1
Score	9	9	9

**Table 9:** Summary of SANRA checklist for narrative reviews. SANRA: Scale for the Assessment of Narrative Review Articles.

	Panchal et al. [21]	Huen et al. [22]	Sokolowska-wojdylo et al. [23]
Justification of the article’s importance	2	2	2
Statement of concrete aims or formulation of questions	2	1	1
Description of the literature search	2	2	2
Referencing	2	2	2
Scientific reasoning	2	1	2
Appropriate presentation of data	1	2	2
Total	11	10	11

Discussion

This systematic review mainly focused on retinoids and their role in the treatment of cutaneous T-cell lymphomas. Our systematic review with its findings, has a significant impact on the future treatment of cutaneous T-cell lymphoma.

*Mechanism of Action*

Retinoids are highly recommended drugs by WHO-EORTC in primary cutaneous T-cell lymphomas. They are derivatives of vitamin A, which mainly modulates cell proliferation, differentiation and immunoregulation of epithelial cells and mononuclear skin infiltrates. Retinoids also induce cellular apoptosis and DNA fragmentation in susceptible T-cells. Retinoids also facilitate the production of gap junctions and glycoproteins, which are lost during the malignant transformation.

The primary effects of retinoids are facilitated by intracellular nuclear receptors: retinoic acid receptors (RARs) and retinoic X receptors (RXRs). They have three subtypes α, β, γ for both the RARs and RXRs receptors. Retinoic acid receptors can homo-dimerize or heterodimerize with RXRs or other nuclear receptors to affect the transcription of genes and produce proteins that affect differentiation and cell growth [23].

Retinoids additionally inhibit ornithine decarboxylase activity, an enzyme that is upregulated in the presence of tumor-promoting substances. First-generation retinoids like alitretinoin, isotretinoin followed by second-generation retinoids like acitretin, etretinate then third-generation analogs like tazarotene and bexarotene are used in the treatment of cutaneous T-cell lymphoma. 

Retinoids in Cutaneous T-cell Lymphomas: First Generation Retinoids

Alitretinoin: Alitretinoin is a synthetically derived first-generation retinoid that can bind to both RAR and RXR; hence, it’s called a pan-retinoic receptor agonist. Alitretinoin exhibits a safety profile compared to other vitamin A derivatives. Two retrospective studies were conducted in 2021 to evaluate the efficacy and tolerability of alitretinoin in patients suffering from mycosis fungoides (MF) and Sezary syndrome (SS).

Alhusayen et al. 2021 [9], from three Canadian centers over five years, studied 48 patients with daily oral dosing of alitretinoin between 10 and 30 mg. In this study, 40% of patients achieved a complete/partial response, 47.5% with stable disease, and 12.5% experienced disease progression. Additionally, 64.3% of patients did not report any side effects. Similarly, Kaemmerer et al. 2021 [10] studied 35 patients with MF and SS. The overall response to alitretinoin was 37.2%, with 28.6% having a stable course and 34.3% experiencing progression. In this study, 63% did not report any side effects, with hypertriglyceridemia being the most common. Cumulatively, these studies suggest alitretinoin is an effective and well-tolerated treatment option for MF and SS, with a significant proportion of patients achieving disease control and a majority not experiencing side effects.

Isotretinoin: Isotretinoin was the first off-label treatment used for cutaneous T-cell lymphoma. Isotretinoin also displayed an antiproliferative and antineoplastic activity, but there is not enough evidence to prove that. Amity-Laish et al. 2019 [11] conducted a retrospective study using acitretin and isotretinoin in 35 patients. The overall response rate was 64% with acitretin and 80% with isotretinoin, but the complete response rate was low at 4% and 8%, respectively. More studies are required to explain the efficacy of isotretinoin.

Second Generation Retinoids

Acitretin: Cheeley et al. 2012 [12] conducted a retrospective study to determine the efficacy and tolerability of acitretin with 32 patients, in which the number of MF (n=29) and SS (n=2). Six patients received acitretin alone, and 26 received acitretin with other cutaneous T-cell lymphoma therapy. The overall response rate was 59%, with a mean duration time of 28 months. Minimal side effects, with five patients discontinuing therapy because of them. Roccuzzo et al. 2022 [19] conducted a retrospective study to evaluate the outcome between acitretin and bexarotene with 82 patients treated at different periods in their treatment course. They both displayed a 66.6% to 75% treatment response. This study shows that advanced-stage patients were treated with bexarotene, whereas acitretin was given to older patients and milder disease conditions.

Etretinate: Etretinate is highly lipophilic with a longer half-life of more than 120 days, requiring more than two years to eliminate from the body after completion of treatment. Only a limited number of studies were available to study the efficacy of etretinate. Most of the studies combined the treatment of Interferon alpha with etretinate as tigason. 

Third Generation Retinoids

Tazarotene: Tazarotene is a third-generation retinoid, mostly used as a topical gel as an adjuvant therapy in refractory MF lesions. The efficacy of tazarotene is explained in only a few studies. Apisarnthanarax et al. 2003 [15] conducted an open-label pilot study with 20 patients with early patch/plaque MF with lesions stable/refractory to therapy for at least eight weeks were included. The tazarotene 0.1% gel was applied for 24 months once daily along with continuous use of other medications. By intent to treat analysis, 58% achieved a global improvement. The side effects like peeling, erythema, burning, and tenderness were seen in 16 patients, managed with steroids or by decreasing the frequency of treatment.

Bexarotene: Bexarotene is a synthetic rexinoid that selectively binds to RXRs. It was the first drug approved by the Food and Drug Administration (FDA) to treat cutaneous T-cell lymphoma in 1999. It was licensed in 2002 as 75 mg soft gelatin capsules for cutaneous T-cell lymphoma after Duvic et al. [8] 2001 conducted randomized open-label, multicenter phase 2 and phase 3 control trials to determine the tolerability, safety, and anti-tumor efficacy of bexarotene (Targretin capsules). This study included 18 international cutaneous T-cell lymphoma centers (USA, Canada, Australia, and Europe). The effects of two randomized doses of 6.5 mg/m^2^ per day vs 650 mg/m^2^ per day were evaluated. Later the dose of Bexarotene was adjusted to 300 mg/m^2^ according to treatment response and side effects. The overall response rate in this study was 20%, 54%, and 67% in the 6.5, 300, and 650 mg/m^2^, respectively. The main side effects observed are hypertriglyceridemia, hypercholesterolemia, headache, central hypothyroidism, asthenia, and leukopenia.

Later, Beltran et al. 2009 [16] conducted a retrospective study to compare the efficacy of bexarotene alone and in combination for treating cutaneous T-cell lymphoma. The overall response for the entire group was 69.2%. Regarding therapy, BEX alone achieved 66%, BEX/IFN showed 40%, and BEX/phototherapy showed 100% with a 20% complete response.

Sokołowska-Wojdyło et al. 2016 [13] and Rouanet et al. 2022 [14] conducted two different retrospective studies with normal doses of 300 mg/m^2^ and at lower doses (<300 mg/m^2^), respectively. The overall response rate with 300 mg/m^2^ is 81%, with side effects like hypertriglyceridemia, hypothyroidism, and a bleeding gastric ulcer. At lower doses, interestingly, the efficacy of bexarotene is not hindered and is also found to be effective when used as the first systemic therapy for the patient. Contrary to the European Organization for Research and Treatment of Cancer (EORTC) recommendations, while using lower doses, there is no requirement to add lipid-lowering agents, but it should be commenced when dyslipidemia is encountered.

In 2013, two studies by Quereux et al. [17] and Weichenthal et al. [18] provided information regarding the long-term tolerability of bexarotene in cutaneous T-cell lymphoma patients and the response of rare variants of cutaneous T-cell lymphoma with bexarotene, respectively. The longest duration of treatment observed was 65.2 months, with 10 patients treated for more than 24 months. The overall response rate was 60% in all patients. Quereux et al. confirm bexarotene is well tolerated for long-term use. Weichenthal et al. state that in classical MF objective response (OR) rate of 37%, other variants of cutaneous T-cell lymphoma like CD30+ALCL 50% OR, LyP 60% OR, SS 33% OR. Bexarotene seems to be a reliable and effective treatment also for non-classical MF and cutaneous T-cell lymphoma variants.

In the same way, Hamada et al. 2021 [20] conducted a cohort study on Japanese patients with cutaneous T-cell lymphoma for bexarotene-post-marketing surveillance. Bexarotene has been available in Japan since 2016. There is a substantial difference in response rates and side effects between patients who started with bexarotene at 300 mg/m^2^ and patients who started less than that. There is an increased response rate with combined photo(chemo)therapy. The present study states that starting bexarotene at 300 mg/m^2^ and a combination of photo(chemo)therapy has positive outcomes.

Limitations

It is vital to consider the limitations of our review. Our systematic review mainly consists of retrospective studies with small sample sizes that could influence the results' validity. Additionally, our studies predominantly include open-access articles, published in English, resulting in a limited number of articles. This overlooks the significant articles published in other languages. The restricted access to articles could result in the omission of critical research papers. While our analysis demonstrated promising outcomes in the clinical studies, methodological limitations and potential biases prevent us from conclusively determining the effectiveness of retinoids in treating cutaneous T-cell lymphoma. Despite these limitations, our systematic review provides valuable insights into treating cutaneous T-cell lymphoma with retinoids and their analogs at different stages. Our study promotes further research into this area for treating cutaneous T-cell lymphoma more efficiently.

## Conclusions

In conclusion, our systematic review demonstrates the role of retinoids and their analogs in treating cutaneous T-cell lymphoma. Our systematic review indicates that Bexarotene has superior outcomes to other retinoids in treating cutaneous T-cell lymphoma. Minimal side effects are observed with all retinoids, the most common being hypertriglyceridemia and hypothyroidism, which can be managed with appropriate therapy. Alitretinoin is the second most common drug, mostly used in the treatment of cutaneous T-cell lymphoma, which has fewer side effects. Retinoids have shown better results when combined with Interferon-α, extracorporeal photopheresis, psoralen plus ultraviolet (PUVA), and total skin electron beam therapy. However, the mechanism of action of retinoids is unclear. Given the limited research available, future studies should focus on large-scale randomized clinical trials to evaluate the efficacy, mechanism of action, and tolerability of retinoids alone and in combination with other systemic therapies.
